# Integrated Analysis of Basic Helix Loop Helix Transcription Factor Family and Targeted Terpenoids Reveals Candidate *AarbHLH* Genes Involved in Terpenoid Biosynthesis in *Artemisia argyi*

**DOI:** 10.3389/fpls.2021.811166

**Published:** 2022-01-17

**Authors:** Xiaozhe Yi, Xingwen Wang, Lan Wu, Mengyue Wang, Liu Yang, Xia Liu, Shilin Chen, Yuhua Shi

**Affiliations:** ^1^Key Laboratory of Beijing for Identification and Safety Evaluation of Chinese Medicine, Institute of Chinese Materia Medica, China Academy of Chinese Medical Sciences, Beijing, China; ^2^School of Chemistry, Chemical Engineering and Life Sciences, Wuhan University of Technology, Wuhan, China

**Keywords:** *Artemisia argyi*, bHLH family, gene expression, terpenoid biosynthesis, 1, 8-cineole, β-caryophyllene

## Abstract

*Artemisia argyi* is a valuable traditional medicinal plant in Asia. The essential oil from its leaves is rich in terpenoids and has been used to enhance health and well-being. In China, the market scale of industries related to *A. argyi* has attained tens of billions of Chinese Yuan. The basic helix-loop-helix (bHLH) family is one of the largest transcription factors families in plants that plays crucial roles in diverse biological processes and is an essential regulatory component of terpenoid biosynthesis. However, the bHLH TFs and their regulatory roles in *A. argyi* remain unknown. Here, 53 *AarbHLH* genes were identified from the transcriptome of *A. argyi* and were classified into 15 subfamilies based on the classification of bHLH proteins in *Arabidopsis thaliana*. The MEME analysis showed that the conserved motif 1 and motif 2 constituted the most conserved bHLH domain and distributed in most AarbHLH proteins. Additionally, integrated analysis of the expression profiles of *AarbHLH* genes and the contents of targeted terpenoids in different tissues group and JA-treated group were performed. Eleven up-regulated *AarbHLHs* and one down-regulated *AarbHLH* were screened as candidate genes that may participate in the regulation of terpenoid biosynthesis (*TPS-AarbHLHs*). Correlation analysis between gene expression and terpenoid contents indicated that the gene expression of these 12 *TPS-AarbHLHs* was significantly correlated with the content changes of 1,8-cineole or β-caryophyllene. Protein–protein interaction networks further illustrated that these *TPS-AarbHLHs* might be involved in terpenoid biosynthesis in *A. argyi*. This finding provides a basis to further investigate the regulation mechanism of *AarbHLH* genes in terpenoid biosynthesis, and will be helpful to improve the quality of *A. argyi*.

## Introduction

*Artemisia argyi* is a valuable traditional medicinal plant and has been widely used in China and other Asian counties. The dried leaf of *A. argyi* is a commonly used Chinese herb called Folium Artemisia Argyi (FAA, Ai Ye). In the Chinese pharmacopeia, FAA is recorded as warming the meridian, arresting bleeding, dispersing cold, and relieving pain (Chinese Pharmacopoeia Commission, 2020). *A. argyi* leaves contain various chemical compositions, including volatile oils, flavonoids, tannins, polysaccharides, etc. (Song et al., 2019). *A. argyi* essential oil (AAEO) is the main active component isolated from FAA. Modern pharmacological studies have shown that AAEO possesses antibacterial, antioxidant, antitumor, analgesic, antiasthmatic, and immunoregulatory activities (Huang et al., 2012; Chen et al., 2017), and it can be helpful for the treatment of various respiratory disorders, such as cough, phlegm, wheezing, and allergies. Additionally, AAEO is also a kind of excellent natural industrial additive used as a natural antimicrobial, pesticide, and insect repellent. Currently, both FAA and AAEO are experiencing increasing demand by the medical and chemical markets. In China, thousands of companies are engaged in the industries related to *A. argyi*, of which more than a dozen have an annual output value of more than 100 million Chinese Yuan, and the overall industry is worth tens of billions of yuan (China Report Net, 2021). Chemical analysis indicated that the main effective components of AAEO were monoterpenes, sesquiterpenes, and their derivatives, for instance, 1,8-cineole, β-caryophyllene, camphor, α-terpineol, thujone, caryophyllene oxide, borneol, etc. (Xiang et al., 2018). Therefore, the study on the regulation metabolism of terpenoid biosynthesis will help improve the quality of *A. argyi.* 1,8-cineole and β-caryophyllene are representative monoterpenes and sesquiterpenes in *A. argyi*, respectively. They are rich in AAEO and have positive pharmacological activities. All terpenoids are biosynthesized from isoprenyl diphosphate formed by five-carbon building blocks isopentenyl diphosphate (IPP) and its isomer dimethylallyl diphosphate (DMAPP), derived from two alternative pathways: the mevalonate (MVA) pathway in the cytosol and the methylerythritol phosphate (MEP) pathway in the plastids (Supplementary Figure S1). The primary precursor of monoterpenes is geranyl diphosphate (GPP, C10), produced through the methylerythritol phosphate (MEP) pathway. GPP is converted into 1, 8-cineole by *TPS-Cin*, one of the downstream terpenoid synthase genes (TPS) (Wise et al., 1998). The precursor farnesyl diphosphate (FPP, C15), biosynthesized from the Mevalonic acid (MVA) pathway, was modified by *TPS21* to form β-caryophyllene (Hong et al., 2012). Transcription factors (TFs) are critical controllers of functional gene expression in these pathways. In plants, five TF families (AP2/ERF, bHLH, MYB, WRKY, and bZIP) have been reported to be involved in the biosynthesis of terpenoids (Lu et al., 2016; Wang et al., 2021).

The basic helix-loop-helix (bHLH) is one of the largest TF families in plants (Heim et al., 2003; Toledo-Ortiz et al., 2003). The highly conserved bHLH domain comprises about 50–60 amino acids and has two functional domains, the basic domain and helix-loop-helix (HLH) domain. The basic region is located at the N-terminal and consists of 15–20 amino acids, usually including five and six basic residues, which participate in DNA binding. The HLH region located at the C-terminus, contains two amphipathic α-helices separated by loop regions, and forms a homodimer or heterodimer to promote protein-protein interaction. The HLH protein without the basic region can forms a dimer with bHLH proteins but lack DNA-binding ability. In plants, the bHLH TFs play critical roles in diverse biological processes (Zhang et al., 2014). Genome-wide comprehensive and systematic analyses of bHLH proteins have been well conducted in Arabidopsis (Toledo-Ortiz et al., 2003), rice (Li et al., 2006), tomato (Sun et al., 2015), and other plant species. It has been reported that bHLH TFs are closely related to the biosynthesis of terpenoids in several plants. For example, in *Arabidopsis thaliana*, the bHLH TF *MYC2* binds to the promoter regions of *TPS21* and *TPS11* synthase genes that catalyze the formation of sesquiterpenes, activates their expression, thereby enhancing the release of sesquiterpenes (Hong et al., 2012). *MYC2* is also indispensable in the regulation of tanshinone and phenolic acid biosynthesis in *Salvia miltiorrhiza* (Zhou et al., 2016). In *Artemisia annua*, AabHLH1 protein binds to the E-box *cis*-elements in the promoters of both ADS and CYP71AV1 genes in the artemisinin biosynthetic pathway and regulates artemisinin biosynthesis (Ji et al., 2014). Overexpression of bHLH iridoid synthesis 1 (*BIS1*) can promote the production of monoterpene indole alkaloids in *Catharanthus roseus* (Van Moerkercke et al., 2015). It is also known that many bHLH family genes involved in the regulation of terpenoid biosynthesis are associated with JA signaling pathway (Chen X. et al., 2019). For example, TcMYC in *Taxus chinensis* was reported as a key regulator of the jasmonic acid (JA) signaling pathway and could bind to the G-box of the promoter of taxane 5α-hydroxylase (Yang et al., 2018). Sui et al. found that overexpression of the bHLH TF *MYC2a* can up-regulate the expression of cembranoid biosynthesis genes, thereby promoting the production of cembranoid in *Nicotiana tabacum* species (Sui et al., 2018).

However, the biosynthesis mechanism of terpenoids in *A. argyi* remains obscure, and the bHLH family of *A. argyi* has not been reported. In this study, we will identify the bHLH family genes in *A. argyi* from its transcriptome data, and analyze their protein physicochemical properties, classification, conservative motif distribution and gene function annotations. Additionally, the differential gene expression analysis, homologous comparison, and correlation analysis between gene expression and terpenoid content will be performed to screen candidate *AarbHLHs* involved in terpenoid biosynthesis in *A. argyi*. It will provide a basis for us to understand the regulation mechanism of AarbHLH TFs in terpenoid biosynthesis in *A. argyi*.

## Materials and Methods

### Material

The plant *Artemisia argyi* used in this study is a widely-grown local variety from Zhangbang Town, Qichun County, Hubei Province, China, which is the genuine producing area of FAA (Ai Ye), also known as Qi Ai. The fresh rhizomes were transplanted into the greenhouse to reproduce seedlings of *A. argyi*. The greenhouse conditions were 14 h day/10 h night photoperiod, the temperature of 25°C (day/night), and 40% relative humidity. Ninety days old seedlings reproduced from a single plant was selected as experimental materials. Different tissues including leaves, stems, and roots were sampled as the LSR group. Leaves were sprayed with 100 μM methyl jasmonate (MeJA) solution (pure water containing 0.098% anhydrous ethanol as the solvent) and were collected after 12 h spraying as the MeJA group, while the same leaves sprayed with empty solvent were harvested as the control group. Three seedlings with uniform growth were selected as a biological replicate, and each experiment was replicated thrice. All samples were frozen in liquid nitrogen and preserved at –80°C for further use.

### RNA-seq Data Information

Three RNA-seq data of *A. argyi* were used in this study. The RNA-seq data of the mixed sample (leaves, stems, and roots) and the MeJA group were completed by our lab, and have been deposited in NCBI (PRJNA785536). The RNA-seq data of the LSR group were obtained from NCBI SRA database (SRP115098), which was completed by Liu et al. (2018).

### Identification of Basic Helix Loop Helix Family Genes in *Artemisia argyi*

The transcriptome of the mixed samples was used to predict the *bHLH* family genes based on the ITAK (Zheng et al., 2016) database^1^. The open reading frames of the candidate genes were predicted by ORFfinder^2^ and translated into amino acid sequences using standard codon translation tables. The Hidden Markov Model (HMM) file corresponding to the bHLH domain (HLH: PF00010) by Pfam (Mistry et al., 2021)^3^ was then used to verify the conserved domain and remove the false positive sequences.

### Phylogenetic Tree Analysis and Protein Domain Analysis of the AarbHLH Proteins

The sequences of *bHLH* genes and proteins in *Arabidopsis thaliana* were downloaded from TAIR. Phylogenetic tree of bHLHs from *A. argyi* and *Arabidopsis thaliana* was constructed by ClustalW and MEGA7 (Kumar et al., 2016) using the neighbor-joining method (Bootstrap repeats = 1000) and visualized by the Figtree V1.4.3 software. The bHLH domain of AarbHLHs was visualized in Jalview (Waterhouse et al., 2009). The conserved motifs were identified by MEME (Bailey et al., 2009)^4^ (motif parameter was set to 10) and visualized by TBtools (Chen et al., 2020a).

### Bioinformatic Analysis

The protein physicochemical properties were calculated by ExPASy ProtParam (Duvaud et al., 2021)^5^. Subcellular localization was predicted using the WoLF PSORT (Horton et al., 2007)^6^. Gene Ontology (GO) functional annotations were conducted using the KOBAS (Bu et al., 2021)^7^. GO is divided into three ontologies: biological processes (BP), molecular function (MF), and cellular components (CC). The top 20 functional terms for credibility in the three categories were selected for visualization (The smaller the *p*-value, the more reliable). Kyoto Encyclopedia of Genes and Genomes (KEGG) annotations were completed using KASS (Moriya et al., 2007)^8^. Homology comparison was completed using the Basic Local Alignment Search Tool (BLAST). Protein sequences of bHLHs related to terpenoid biosynthesis were downloaded from NCBI^9^. Protein–protein interaction networks were predicted using STRING.4^10^ (Szklarczyk et al., 2019) based on *Arabidopsis thaliana* homologous proteins.

### Content Determination of 1,8-Cineole and β-Caryophyllene

The pulverized plant material (1.0 g) from the LSR group and the MeJA group were accurately weighed and sequentially refluxed for 90 min with 10 mL hexane, then the extract was separated by filtration.

The extractives content of 1, 8-cineole and β-caryophyllene were determined using Gas Chromatography/Mass Spectrometry (GC-MS) (Chen et al., 2020b). The chromatographic column was HP - 5MS UI capillary column. Chromatographic conditions were as follows: programmed temperature, the initial temperature of 40°C, maintained for 2.5 min; It was increased to 200°C at a rate of 5°C⋅min^–1^ and maintained for 1 min, then increased to 240°C at 10°C⋅min^–1^ and maintained for 5 min. The carrier gas was helium, and the injector temperature was 250°C, and the splitless injection volume was 1 μL. Mass spectrum conditions: EI ionization source, electron energy 70 eV, temperature 250°C, mass scanning range m/z 35–550. The identification of 1, 8-cineole, and β-caryophyllene was based on comparing their mass spectra with the mass spectra library (from NIST resources) and retention times with standards. Both 1, 8-cineole and β-caryophyllene standards were obtained from ChemFaces (Wuhan, China).

### Expression Profiles Analysis of *AarbHLHs* and Real-Time Quantitative PCR Verification

The gene expression profiles of *AarbHLHs* were analyzed based on Fragments Per Kilobase of Exon model Per Million mapped Fragments (FPKM). Expression heat maps were generated using TBtools software.

Total RNA was extracted from the LSR group and the MeJA group by EasyPure^®^Plant RNA Kit (ER301-01, Transgen Biotech), and then reverse-transcribed into cDNA using the Transscript^®^One-Step gDNA Removal and cDNA Synthesis Supermix kit (AT311-02, Transgen Biotech). Additionally, Rotor-Gene Q real-time quantitative fluorescence PCR instrument (QIAGEN) and TransStart Green qPCR Supermix UDG kit (AQ111-01, Transgen Biotech) were used in the Quantitative PCR (qRT-PCR) experiment. qPCR primers were designed using Primer Premier 5.0 (Supplementary Table S1). *Actin* was used as the internal reference gene correction result (Yi et al., 2021). The qRT-PCR system was 20 μL: 2 × Transstart TipTop Green qPCR SuperMix 10 μL, upstream primers (10 μm) 0.2 μL, downstream primers (10 μm) 0.2 μL, cDNA 1.0 μL, and ddH2O 8.6 μL. The preparation process was completed on ice. The PCR amplification procedure was as follows: pre-denaturation at 94°C for 10 min; 95°C, 10 s, 60°C, 15 s, 72°C, 20 s, 40 cycles; Melting curve analysis program: 95°C, 15 s, 60°C, 60 s, 95°C, 15 s. Each sample was set with three technical replicates and negative control. 2–^ΔΔCT^ method was used to calculate the relative expression levels. The results were expressed as mean ± SD over three replicates.

### Correlation Analysis

The correlations between the expression of *TPS-AarbHLHs* in qRT-PCR and the content of 1,8-cineole and β-caryophyllene were analyzed by the Pearson correlation method with SPSS software.

## Results

### Identification and Characterization of Basic Helix Loop Helix Family Genes in *Artemisia argyi*

Sixty-three bHLH sequences were generated from the transcriptome data using ITAK. After removing redundant and false sequences using HMM, 53 bHLH proteins were identified and renamed as AarbHLH1∼AarbHLH53. The length of AarbHLH proteins varied from 89 to 625 amino acids, the predicted molecular weights ranged from 10.11 to 68.59 kDa, and the predicted isoelectric points ranged from 4.73 to 9.93. All AarbHLH proteins are likely hydrophilic (GRAVY < 0). Two bHLH proteins (AarbHLH48 and AarbHLH26) were stable proteins (Instability index < 40), while others were classified as unstable proteins. The aliphatic index ranged from 44.52 to 96.49. The prediction of subcellular localization proposed that almost all AarbHLH proteins were probably localized into the nucleus, and a few protein sequences may be located in the cytoplasm and chloroplast (Table 1).

**TABLE 1:** The complete list of AarbHLH proteins and their physiochemical properties.

Protein ID	Unigene ID	Amino acid length	PI	MW (Da)	GRAVY	Instability index	Fatty coefficient	Subcellular localization
AarbHLH1	Cluster-17295.0	172	4.73	18999.31	–0.433	44.44	78.20	Nuclear
AarbHLH2	Cluster-17885.0	185	8.98	20688.00	–0.340	58.49	96.49	Cytoplasmic
AarbHLH3	Cluster-22634.0	234	5.59	26344.08	–0.501	47.34	79.19	Cytoplasmic
AarbHLH4	Cluster-23024.0	301	7.03	33525.73	–0.828	43.53	59.53	Nuclear
AarbHLH5	Cluster-23094.0	93	8.98	10271.60	–0.441	61.42	89.25	Chloroplast
AarbHLH6	Cluster-23287.0	425	6.43	47426.44	–0.754	56.23	44.52	Nuclear
AarbHLH7	Cluster-23366.0	503	6.37	55301.33	–0.745	64.91	54.87	Nuclear
AarbHLH8	Cluster-24016.0	387	8.54	41657.34	–0.662	71.46	51.21	Chloroplast
AarbHLH9	Cluster-24487.0	346	6.33	38949.94	–0.524	61.26	78.09	Nuclear
AarbHLH10	Cluster-24526.0	240	6.31	26382.63	–0.588	54.04	70.75	Nuclear
AarbHLH11	Cluster-25306.0	503	6.27	56549.95	–0.530	48.11	77.30	Nuclear
AarbHLH12	Cluster-25795.0	121	9.61	13853.96	–0.631	44.79	71.74	Nuclear
AarbHLH13	Cluster-26014.0	253	6.55	28299.83	–0.724	55.69	56.32	Nuclear
AarbHLH14	Cluster-26181.0	562	6.44	61282.00	–0.596	51.71	63.95	Nuclear
AarbHLH15	Cluster-26396.1	450	7.69	48895.81	–0.690	46.88	54.00	Nuclear
AarbHLH16	Cluster-26909.0	292	9.23	31102.44	–0.393	45.92	75.92	Chloroplast
AarbHLH17	Cluster-27334.0	249	8.85	27022.05	–0.663	54.60	59.56	Nuclear
AarbHLH18	Cluster-27352.0	234	6.85	25933.42	–0.700	54.94	68.42	Nuclear
AarbHLH19	Cluster-27761.0	312	6.47	35381.54	–0.537	55.11	78.40	Nuclear
AarbHLH20	Cluster-28036.0	502	5.53	53659.02	–0.724	57.46	57.73	Nuclear
AarbHLH21	Cluster-28463.0	567	7.00	62228.30	–0.781	60.56	64.16	Nuclear
AarbHLH22	Cluster-29711.0	141	5.15	16356.21	–0.896	65.21	66.45	Nuclear
AarbHLH23	Cluster-30109.0	283	5.21	31534.64	–0.553	54.40	82.93	Nuclear
AarbHLH24	Cluster-30325.17	209	8.95	23334.70	–0.735	49.55	69.14	Nuclear
AarbHLH25	Cluster-30469.0	173	6.11	19004.36	–0.621	61.05	74.45	Nuclear
AarbHLH26	Cluster-30602.0	185	8.51	21037.10	–0.434	37.93	90.11	Cytoplasmic
AarbHLH27	Cluster-31195.0	291	5.36	32087.85	–0.597	58.32	72.47	Nuclear
AarbHLH28	Cluster-31591.0	323	8.16	36296.67	–0.785	54.25	63.10	Nuclear
AarbHLH29	Cluster-31772.0	292	6.31	32393.06	–0.808	41.92	64.38	Nuclear
AarbHLH30	Cluster-32521.0	224	5.31	25340.65	–0.797	56.22	66.74	Nuclear
AarbHLH31	Cluster-33144.0	225	5.61	25492.78	–0.631	62.24	74.44	Nuclear
AarbHLH32	Cluster-33296.0	223	8.43	24512.54	–0.640	43.42	76.05	Nuclear
AarbHLH33	Cluster-34323.0	272	7.79	30624.94	–0.436	47.33	81.36	Nuclear
AarbHLH34	Cluster-34901.0	308	6.19	34078.60	–1.022	60.08	50.94	Nuclear
AarbHLH35	Cluster-35435.0	128	9.93	14475.62	–0.615	50.04	69.30	Cytoplasmic
AarbHLH36	Cluster-35828.0	237	8.28	26493.96	–0.788	54.76	58.44	Nuclear
AarbHLH37	Cluster-36026.0	260	5.77	28290.40	–0.816	60.11	56.23	Nuclear
AarbHLH38	Cluster-36269.0	305	6.37	33640.71	–0.994	54.51	63.90	Nuclear
AarbHLH39	Cluster-37717.0	399	5.78	44487.75	–0.713	46.04	60.13	Nuclear
AarbHLH40	Cluster-38739.0	414	9.03	46694.13	–1.096	56.03	49.06	Nuclear
AarbHLH41	Cluster-38842.0	89	9.03	10107.37	–0.725	90.20	96.40	Nuclear
AarbHLH42	Cluster-39067.0	256	8.32	28746.71	–0.641	46.59	75.04	Nuclear
AarbHLH43	Cluster-39263.0	363	6.09	41115.20	–0.947	65.81	54.85	Nuclear
AarbHLH44	Cluster-40318.0	472	6.22	52407.20	–0.511	41.79	77.90	Nuclear
AarbHLH45	Cluster-40688.0	444	9.30	49004.33	–0.560	41.70	73.11	Nuclear
AarbHLH46	Cluster-40751.0	481	5.92	53153.34	–0.717	50.21	60.17	Nuclear
AarbHLH47	Cluster-40894.0	625	5.09	68586.91	–0.515	44.56	74.05	Nuclear
AarbHLH48	Cluster-41025.0	418	8.67	45937.65	–0.631	32.85	70.17	Nuclear
AarbHLH49	Cluster-41392.0	226	7.16	25319.03	–0.488	50.63	64.34	Nuclear
AarbHLH50	Cluster-42010.0	271	6.76	30469.19	–0.582	56.02	60.74	Nuclear
AarbHLH51	Cluster-42234.0	369	5.95	41799.27	–0.899	56.73	60.24	Nuclear
AarbHLH52	Cluster-42604.0	288	5.51	30493.20	–0.369	46.50	76.91	Nuclear
AarbHLH53	Cluster-9454.0	285	6.40	32180.39	–0.501	68.27	76.63	Nuclear

*Information provided includes gene names, gene Ids, theoretical subcellular localization, protein length, molecular weight (MW; Da), Instability index, fatty coefficient, and theoretical isoelectric points (PI). MW, molecular weight; PI, theoretical isoelectric point; GRAVY, grand average of hydropathicity.*

### Phylogenetic Analysis of the AarbHLH Family Proteins

**FIGURE 1:**
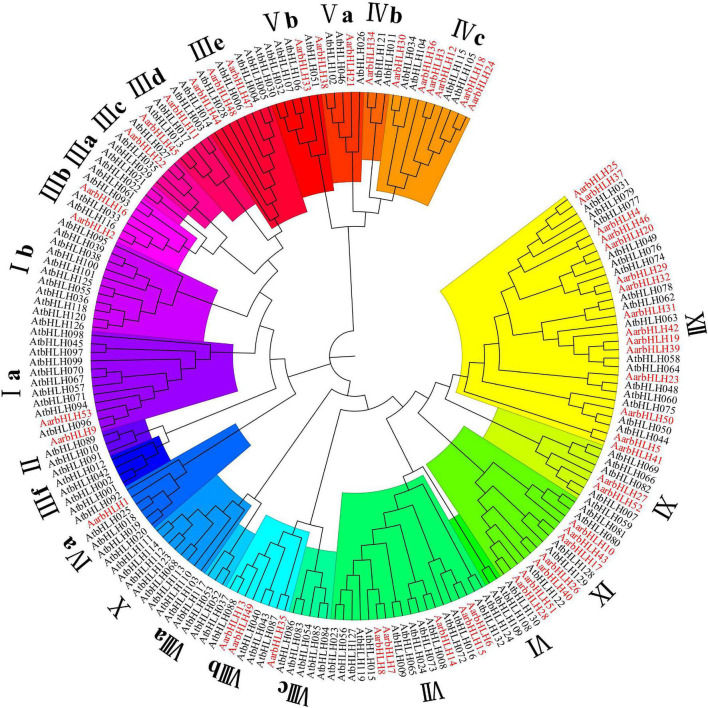
The phylogenetic tree of bHLH proteins in *Artemisia argyi* and *Arabidopsis thaliana.* The tree was constructed using MEGA 7.0 with the Neighbor Joining-NJ method (bootstrap values for 1000 replicates). The colored branches indicate different subgroups. Red fonts represent AarbHLH proteins. Black fonts represent AtbHLH proteins.

To classify these AarbHLH proteins, a phylogenetic tree was constructed based on 53 AarbHLH protein sequences in *A. argyi* and 128 AtbHLH protein sequences in *Arabidopsis thaliana* (Figure 1). It is shown that the AarbHLH proteins were classified into 15 subfamilies (Ia, IIIb, IIIc, IIId, IIIe, IVa, IVb, IVc, Va, Vb, VII, VIIIb, IX, XI, and XII) according to the classification of the bHLH proteins in *Arabidopsis thaliana* and no AarbHLH protein was not classified. The subfamily XII was the largest group in *A. argyi* (15 proteins) and *Arabidopsis thaliana* (16 proteins). The number of IVb, Vb, IIIc, and IVa subfamily proteins in *A. argyi* was the least, respectively containing only one protein. None of AarbHLH proteins were grouped into subfamilies Ib, II, IIIa, IIIf, VI, VIIIa, VIIIc, X.

### Analysis of Basic Helix Loop Helix Domain and Conserved Motifs of AarbHLH Proteins

**FIGURE 2:**
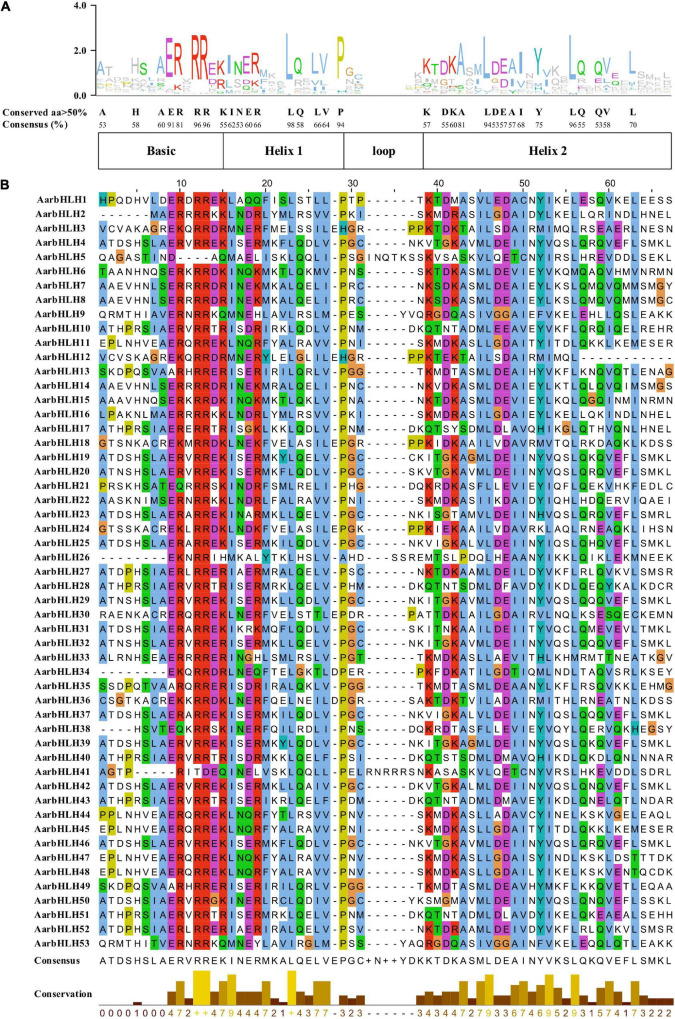
The conserved bHLH domains of AarbHLH proteins in *Artemisia argyi*. **(A)** The sequence logo of the bHLH domain. The capital letters represent over 50% conservation of amino acids. **(B)** The distribution of the conserved bHLH domain in AarbHLH proteins.

The bHLH domain generally contains two functional regions, a basic region and a HLH region. The basic region, located at the N-terminal, consists of 10–15 amino acids and functions as a DNA binding motif. The HLH region located at the C-terminal is promoting the formation of homodimers or heterodimers. Amino acid sequence comparison of AarbHLH proteins showed that 53 AarbHLH proteins possessed a highly conserved bHLH domain, which contained the basic, helix 1, loop, and helix 2 regions (Figure 2). In the basic region, 37 AarbHLH proteins contained His-5/Lys-5, Glu-9, and Arg-13 residues that were proven to bind to G-box (CACGTG); 11 AarbHLH proteins contained Glu-9 and Arg-13 residues, which could bind to the E-box (CANNTG). Three proteins contained only Glu-9 or Arg-13 and recognized non-E-box binding sequences. The remaining two proteins lacked Glu-9 or Arg-13 and did not have the DNA-binding ability. In the HLH region, the conservatism of five amino acid residues (Leu-23, Pro-29, Ala-43, Leu-46, Leu-56) was more than 80%, which was necessary to maintain the stability of the dimer structure. The conservatism of Leu-23 and Leu-56 was 98 and 96%, which proved that they are essential for the formation of the dimer structure.

**FIGURE 3:**
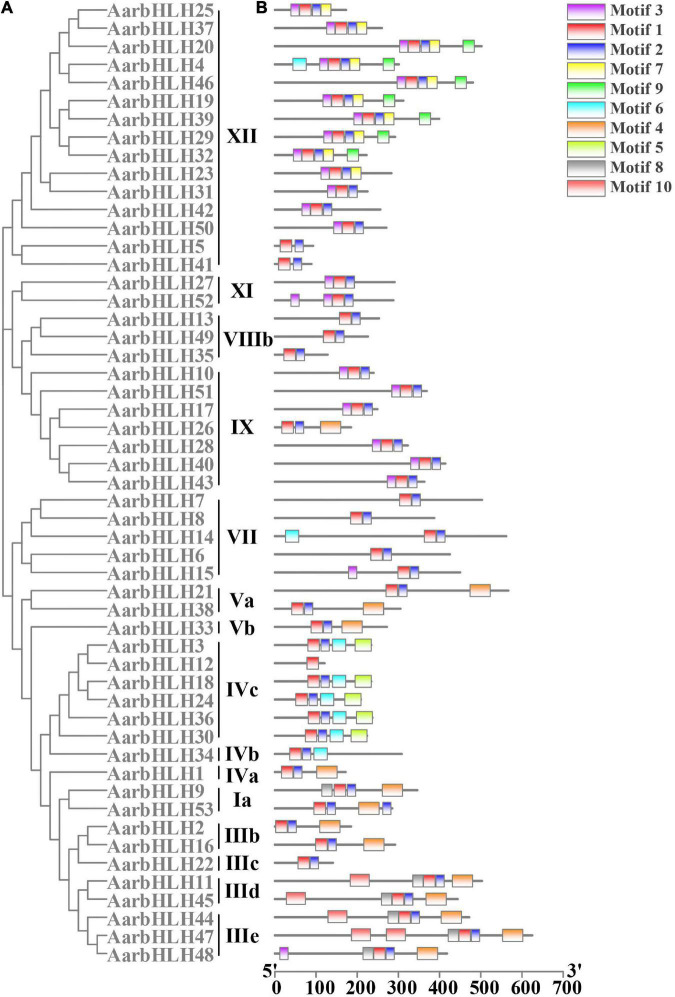
Phylogenetic relationships and conserved motifs analysis of AarbHLH proteins. **(A)** Neighbor-joining phylogenetic tree of AarbHLHs (bootstrap values for 1000 replicates). **(B)** Distribution of conserved motifs in AarbHLH proteins. The colored boxes represent different motifs. The box length represents motif length.

To analyze the functions of AarbHLH proteins, the conserved motifs of AarbHLH proteins were detected using MEME. The result indicated that AarbHLH proteins contained a different number of conserved motifs (Figure 3 and Supplementary Figure S2). All proteins were discovered to have two highly conserved motifs, Motif 1 and Motif 2, located in the bHLH domain except for AarbHLH12. Motif 1 had 29 amino acids, and Motif 2 had 21 amino acids. Motif 1 and Motif 2 were adjacent to each other, but there was a gap in the middle, and Motif 1 was closer to the N-terminal. Visual analysis of the bHLH domain showed that Motif 1 was a combination of the basic region and the helix1 region, Motif 2 was the helix2 region. The gap between Motif 1 and Motif 2 was due to the loop region being very divergent, which did not contain any conserved amino acids. The conserved motif prediction result of MEME and the identification result of the bHLH domain of AarbHLH proteins supported each other and further proved the high reliability of the screening result of the bHLH gene family based on HMM in 3.1. Although AarbHLH12 was not predicted for Motif 2, it was found that it was only missing a portion of the helix2 region in its amino acid sequence but still retains an essentially conserved amino acid Leu-56, as a result it may still perform its normal function. Additionally, the motifs belonging to the same subfamily of AarbHLH proteins were the same or similar. For example, Motifs 1, 2, and 3 were identified in 13 of the 15 members of subfamily XII, and Motifs 1, 2, 5, and 6 were identified in five of the six members of the subfamily IVc. In subfamily III (e + d), Motifs 1, 2, 4, 8, and 10 were identified in four of the five members, with AarbHLH48 lacking only Motif 10. Some motifs were found only in specific subfamilies, suggesting that these motifs may have a particular function. For example, Motif 5 was limitedly present in subfamily IVc, and Motif 7 and Motif 9 were specific to subfamily XII.

### Gene Ontology and Kyoto Encyclopedia of Genes and Genomes Annotation

**FIGURE 4:**
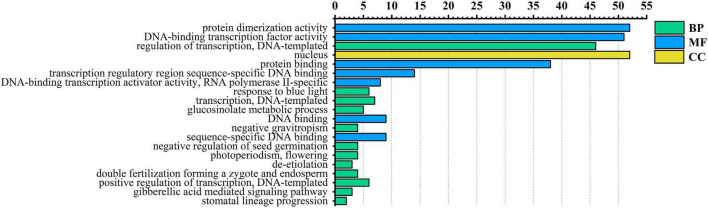
Gene ontology (GO) enrichment analysis of AarbHLH proteins. The top 20 GO terms of level 2 in molecular function (MF), biological process (BP), and cellular component (CC) were visualized. The *x*-axis shows the enriched protein numbers, and the *y*-axis shows the information of GO terms.

To further understand the functions of the AarbHLH proteins, *Arabidopsis thaliana* was used as a model species for GO annotation and GO enrichment analysis. GO annotation indicated 53 AarbHLH proteins were annotated to 76 GO terms, including 56 BP terms, 14 MF terms, and six CC terms. The top 20 GO terms of level two were visualized (Figure 4). In BP terms, it was found that 46 *AarbHLH* genes were involved in the biological regulation. In CC terms, 52 *AarbHLH* genes were components of the nucleus, but few of them were components of the vacuole and cytoplasm. In MF terms, 52 *AarbHLH* genes and 51 *AarbHLH* genes were classified into protein dimerization activity and binding TF activity, respectively.

The KEGG pathway analysis showed that only *AarbHLH14*, *AarbHLH44*, *AarbHLH 47*, *AarbHLH 48* were annotated to two KEGG homologous genes, assigned three pathways. The *AarbHLH14* gene was a homologous gene of *PIF3* (KO: K12126) and participated in plant hormone signal transduction pathway (ko04075) and circadian rhythm – plant pathway (ko04712). *AarbHLH44*, *AarbHLH 47*, *AarbHLH 48* were annotated to *MYC2* (KO: K13422) that participated in plant hormone signal transduction pathway (ko04075) and MAPK signaling pathway (ko04016). Therefore, these four genes are all involved in plant hormone signal transduction. *PIF3* mainly transmits gibberellin (GA) signal, *MYC2* mainly transmits JA signal.

### The Content Changes of 1,8-Cineole and β-Caryophyllene in *Artemisia argyi*

**FIGURE 5:**
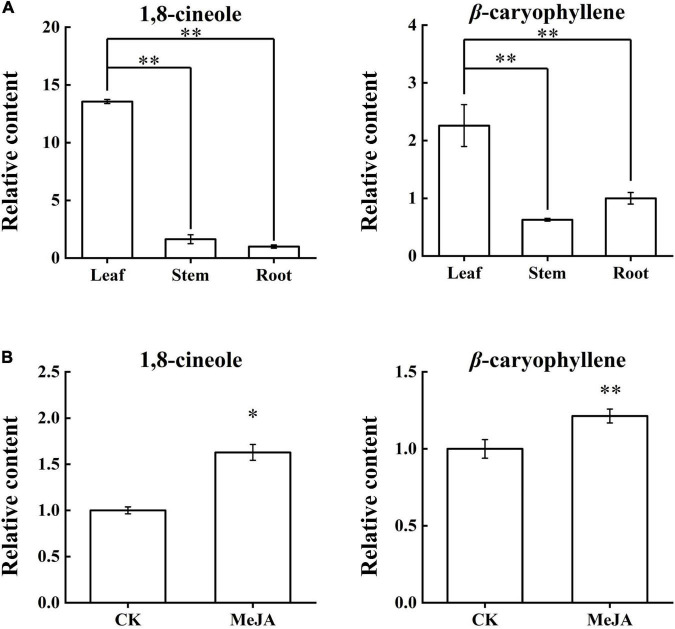
Content changes of 1,8-cineole and β-caryophyllene in the LSR group and the MeJA group. **(A)** Relative contents of 1,8-cineole and β-caryophyllene in the LSR group. **(B)** Relative contents of 1,8-cineole and β-caryophyllene in the MeJA group. The values represent means ± SD (*n* = 3). * Means *p*-value < 0.05, and ^**^ means *p*-value < 0.01.

Chromatography/Mass Spectrometry was used to determine the content changes of 1,8-cineole and β-caryophyllene in the LSR group and the MeJA group (Supplementary Figure S3 and Supplementary Table S2). Content analyses of the LSR group indicated that the contents of 1,8-cineole and β-caryophyllene were the highest in the leaves (Figure 5A). The content of 1,8-cineole in leaves was about 8.3 times higher than that in stems, and 13.5 times higher than that in roots, showed an extremely significant difference (*p-*value < 0.01). Similarly, the content of β-caryophyllene in leaves was about 3.5 times higher than that of stems, and about 2.2 times higher than that of roots, showed an extremely significant difference (*p*-value < 0.01). After MeJA treatment for 12 h, 1,8-cineole content increased by 1.6 times (*p*-value < 0.05), and the content of β-caryophyllene increased by 1.2 times (*p*-value < 0.01) (Figure 5B). In addition to confirming the literature results, this result improved the data for us to further analyze the relationship between compound content and expression profiles of *AarbHLH* genes.

### Gene Expression Profiles of *AarbHLHs* and Candidate *AarbHLHs* Involved in Terpenoid Biosynthesis

**FIGURE 6:**
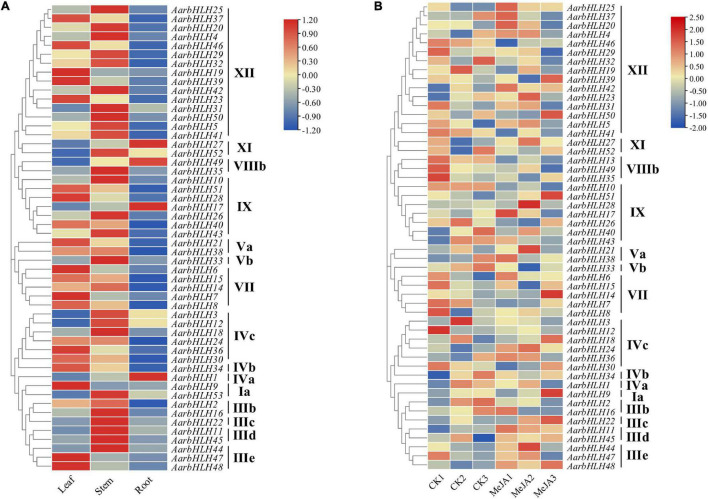
The heat maps of gene expression of *AarbHLHs* in the LSR group and the MeJA group. **(A)** The heat map of *AarbHLHs* expression in the LSR group. **(B)** The heat map of *AarbHLHs* expression in the MeJA group.

The expression profiles of *AarbHLH* genes were analyzed using RNA-seq data of the LSR group and the MeJA group (Figure 6 and Supplementary Table S3). In the LSR group, 52 members of the 53 *AarbHLHs* family genes were expressed at least in one tissue, while *AarbHLH13* was not expressed. *AarbHLHs* were mainly expressed in leaves and stems but less in roots, and *AarbHLHs* of the same subfamily had similar expression patterns. In the MeJA group, 52 *AarbHLH* genes were expressed, while *AarbHLH53* was not expressed. *AarbHLHs* belonging to MYC class (IIIe + d) were all up-regulated under MeJA treatment, which was consistent with the content change trends of terpenoids. The fold change of FPKM > 1.5 was considered as a threshold to screen differentially expressed genes (DEGs) in both groups. In the LSR group, it was revealed that nine *AarbHLH* genes (*AarbHLH6*, *7*, *9*, *19*, *21*, *23*, *28*, *39*, *47*) were highly expressed, and 11 *AarbHLH* genes (*AarbHLH1*, *3*, *11*, *12*, *17*, *22*, *27*, *31*, *49*, *52*, *53*) were lowly expressed in leaves. In the MeJA group, seven *AarbHLH* genes (*AarbHLH11*, *21*, *22*, *24*, *28*, *44*, *48*) were up-expressed and five *AarbHLH* genes (*AarbHLH2*, *10*, *13*, *35*, *52*) were down-expressed. Among them, *AarbHLH21* and *AarbHLH28* were up-regulated, and *AarbHLH52* was down-regulated both in the LSR group and the MeJA group. Thus, *AarbHLH21*, *AarbHLH28*, and *AarbHLH52* were screened as candidate *AarbHLH* genes involved in terpenoid biosynthesis (named TPS-AarbHLH genes) in *A. argyi*, of which *AarbHLH21*, *AarbHLH28* may be positive regulators, and *AarbHLH52* was may be a negative regulator.

**FIGURE 7:**
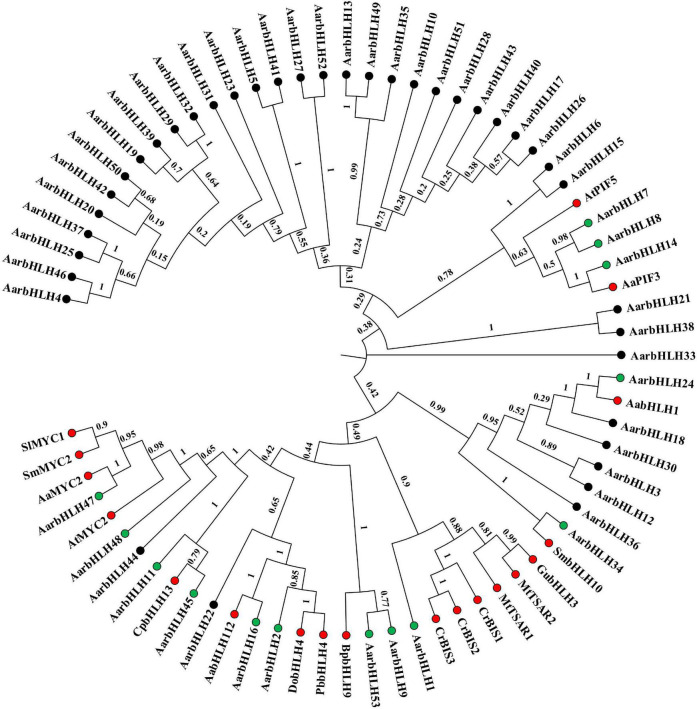
The phylogenetic tree analysis based on the protein sequences of 53 AarbHLHs and nine known TPS-bHLHs by Neighbor-Joining (NJ) tree method. Red dots represent TPS-bHLHs. Green dots represent TPS-AarbHLHs. The number on the branch represents the confidence of the branch.

Literature research found that 18 bHLH genes in other plant species have been reported to play a positive role in terpenoid biosynthesis (named TPS-bHLH genes) (Table 2). Therefore, homology comparison was used to investigate more candidate positive *TPS-AarbHLH* genes in *A. argyi*. The phylogenetic tree analysis based on the protein sequences of 53 AarbHLHs and 19 known TPS-bHLHs indicated that 14 AarbHLH proteins (AarbHLH1, 2, 7, 8, 9, 11, 14, 16, 24, 34, 45, 47, 48, and 53) were clustered in the same branch with these known TPS-bHLH proteins, respectively (Figure 7). The BLAST result showed that the 14 AarbHLH proteins shared high similarity with known bHLHs proteins (Supplementary Table S4). Thus, these 14 *AarbHLH* genes were considered as homologous genes of known TPS-bHLHs, indicating they may also have positive regulatory roles in terpenoid biosynthesis. The expression profiles of these genes in transcriptome data further indicated that six *AarbHLH* genes (*AarbHLH7*, *9*, *11*, *24*, *47*, *48)* were significantly up-regulated in the LSR group or in the MeJA group, four *AarbHLH* genes (*AarbHLH8*, *14*, *16*, *34*) were slightly up-regulated, while *AarbHLH1*, *2*, *45*, and *53* showed some down regulation. Considering that homologous genes are likely to have similar functions, 10 homologous *AarbHLHs* with up-regulated trends were selected to further verify their functional roles.

**TABLE 2:** Nine known bHLH proteins involved in the regulation of terpenoid biosynthetic pathway.

Protein name	Species	GenBank ID	References
AtMYC2	*Arabidopsis thaliana*	AT1G32640	Hong et al., 2012
AtPIF5	*Arabidopsis thaliana*	AT3G59060	Mannen et al., 2014
AabHLH1	*Artemisia annua*	PWA82566	Ji et al., 2014
AabHLH112	*Artemisia annua*	PWA98909.1	Xiang et al., 2019
AaPIF3	*Artemisia annua*	PWA54178.1	Zhang et al., 2019
AaMYC2	*Artemisia annua*	KP119607.1	Shen et al., 2016
SmMYC2	*Salvia miltiorrhiza*	KJ945636	Zhou et al., 2016
SmbHLH10	*Salvia miltiorrhiza*	MH549183	Xing et al., 2018
SlMYC1	*Solanum lycopersicum*	AIC63945.1	Xu et al., 2018
CrBIS1	*Catharanthus roseus*	KM409646	Van Moerkercke et al., 2015
CrBIS2	*Catharanthus roseus*	KM409645.1	Van Moerkercke et al., 2016
CrBIS3	*Catharanthus roseus*	MN646782.1	Singh et al., 2021
PbbHLH4	*Phalaenopsis bellina*	KY979199	Chuang et al., 2018
BpbHLH9	*Betula platyphylla*	KX518840	Yin et al., 2017
GubHLH3	*Glycyrrhiza uralensis*	LC318133	Tamura et al., 2018
CpBHLH13	*Chimonanthus praecox*	RWR96436.1	Aslam M. Z. et al., 2020
DobHLH4	*Dendrobium officinale*	QWS71161.1	Yu et al., 2021
MtTSAR1	*Medicago truncatula*	XP013456259.2	Mertens et al., 2016
MtTSAR2	*Medicago truncatula*	XP013456259.2	Mertens et al., 2016

**FIGURE 8:**
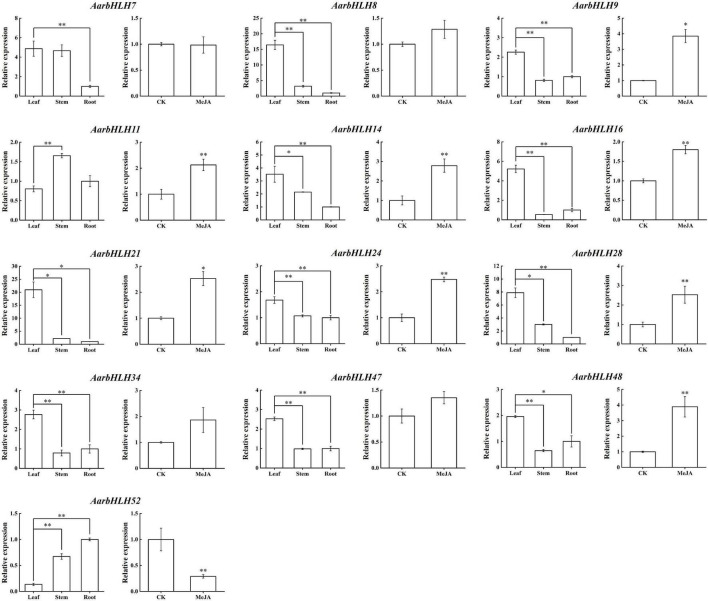
qRT-PCR of candidate *AarbHLHs* for terpenoid synthesis in the LSR group and the MeJA group. The values represent means ± SD (*n* = 3). * Means *p*-value < 0.05, and ^**^ means *p*-value < 0.01.

To summarize, a total of 13 candidate *TPS-AarbHLHs*, including 12 positive *TPS-AarbHLHs* (*AarbHLH7*, *8*, *9*, *11*, *14*, *16*, *21*, *24*, *28*, *34*, *47*, *48*) and one negative *TPS-AarbHLH* (*AarbHLH52*) were screened through combining with gene differential expression analysis and homology comparison analysis. qRT-PCR was further used to verify the expression profiles of the candidate *TPS-AarbHLHs*. The result indicated that 11 *TPS-AarbHLHs* was significantly up-regulated and *AarbHLH52* were significantly down-regulated at least one of the two groups, which were consistent with their expression trends in the RNA-Seq data (Figure 8 and Supplementary Figure S4). But the expression of *AarbHLH7* showed some difference. *AarbHLH7* was highly expressed in leaves than stems and roots in the RNA-seq data of the LSR group, but its expression level in leaves was similar to that of stems in qRT-PCR result. Therefore, 12 *TPS-AarbHLHs* (11 positive regulators and one negative regulator) were finally screened for further study.

### The Correlation Analysis of Candidate *TPS-AarbHLH* Genes and the Content of 1,8-Cineole and β-Caryophyllene

We further used Pearson’s correlation analysis to study the relationship between the expression of candidate TPS-AarbHLH genes and the content changes of target terpenoid compounds (Table 3). The result showed that the expression of seven *TPS-AarbHLHs* was significant correlated with the content of 1,8-cineole and β-caryophyllene in both the LSR group and the MeJA group, of which *AarbHLH21* and *AarbHLH48* showed extremely significant correlation, the expression of four *TPS-AarbHLHs* were significantly correlated with the content of 1,8-cineole and β-caryophyllene in both the LSR group and the MeJA group in one group, while the expression of *AarbHLH11* only exhibited a positive correlation with β-caryophyllene in the MeJA group. In summary, 10 of 12 candidate *TPS-AarbHLH* genes had a significant positive correlation with 1,8-cineole and β-caryophyllene in at least one group, and *AarbHLH 52* had a significant negative correlation with these two terpenoid compounds in the two groups, further demonstrating these 11 *TPS-AarbHLH* genes play roles in the regulation of terpenoids biosynthesis. Additionally, different *TPS-AarbHLH* genes showed different correlations with the target terpenoid compounds, suggesting that these genes may have functional differentiation.

**TABLE 3:** Correlation analysis between the contents of 1,8-cineole and β-caryophyllene and expression of candidate *TPS-AarbHLHs*.

	Correlation coefficient			
*TPS-AarbHLHs*	1,8-cineole		β -caryophyllene	
	LSR	MeJA	LSR	MeJA
*AarbHLH8*	0.986**	0.420	0.918**	0.611
*AarbHLH9*	0.981**	0.794	0.923**	0.907*
*AarbHLH11*	–0.635	0.708	−0.790*	0.833*
*AarbHLH14*	0.861**	0.750	0.650	0.874*
*AarbHLH16*	0.985**	0.812*	0.924**	0.926**
*AarbHLH21*	0.983**	0.938**	0.860**	0.961**
*AarbHLH24*	0.955**	0.885*	0.836**	0.940**
*AarbHLH28*	0.962**	0.948**	0.788*	0.930**
*AarbHLH34*	0.962**	0.936**	0.948**	0.858*
*AarbHLH47*	0.990**	0.765	0.950**	0.739
*AarbHLH48*	0.922**	0.967**	0.926**	0.964**
*AarbHLH52*	−0.935**	−0.872*	−0.787*	−0.927**

*Means p-value < 0.05, and ** means p-value < 0.01.

### Protein–Protein Interaction Networks of Candidate TPS-AarbHLH Proteins

**FIGURE 9:**
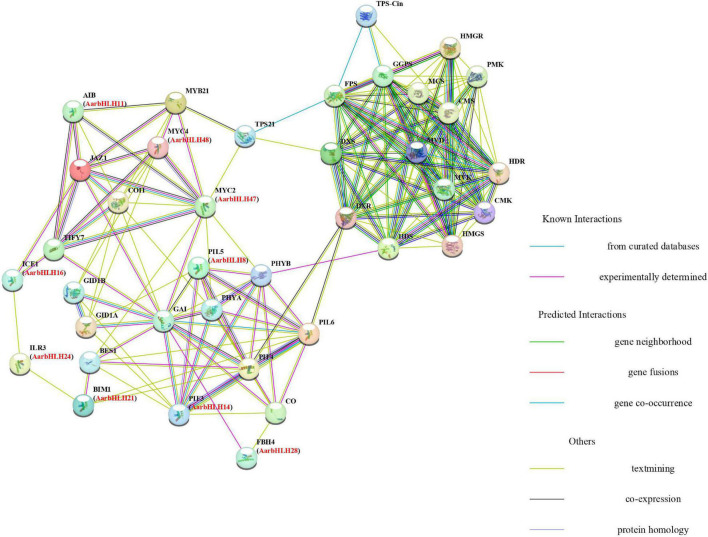
Protein–protein interaction networks analysis between candidate TPS-AarbHLHs and terpenoid biosynthesis enzyme proteins using *Arabidopsis thaliana* orthologs. Predictions were completed using STRING 11, and candidate TPS-AarbHLHs were listed in brackets with their *Arabidopsis thaliana* orthologs.

The protein–protein interaction (PPI) networks (Figure 9) of the 12 TPS-AarbHLH proteins with terpenoid biosynthesis-related enzyme proteins were predicted using STRING to explore their possible regulatory mechanism in terpenoids biosynthesis. The homologous proteins of TPS-AarbHLH in Arabidopsis were listed in Supplementary Table S5. The result revealed that nine TPS-AarbHLH proteins (AarbHLH8, 11, 14, 16, 21, 24, 28, 47, and 48) constituted an entire interaction network. AarbHLH8, 14, 21, 24, along with PHYA, PHYB, CO, and other proteins, comprised a part of the optical signal transmission network. The AarbHLH28 protein participated in the transmission of GA signal by interacting with GAI, GID1A, GID1B, and other GA signaling proteins. AarbHLH47 and 48 were MYC proteins, and AarbHLH16 was an MYC-like protein, which were important TFs in the JA signaling network. AarbHLH11 is an ABA-inducible bHLH TF that interacts with multiple factors in the JA network. Several signaling proteins in the three protein networks interact with proteins in the other two networks, thus establishing a link between them. For example, AarbHLH14 (homology ID: PIF3) and AarbHLH8 (homology ID: PIL5) interacted with the GA signaling protein GAI in the optical signal network. AarbHLH28 (homology ID: FBH4) interacted with light signal proteins and GA signaling proteins. GA signaling proteins GID1A and GID1B interacted with AarbHLH48 (homologous ID: MYC4). AarbHLH16 (same ID: ICE1), AarbHLH21 (same ID: BIM1) and AarbHLH24 (same ID: PIF3) established a bridge between JA signal and optical signal. In this group, AarbHLH47 (homology ID: MYC2) was the key factor of the optical signal, GA signal, and JA signal and played an important role in multiple signal transmission. According to the prediction of interaction networks between candidate TPS-AarbHLH proteins and terpenoid biosynthesis enzyme proteins, candidate TPS-AarbHLHs played a direct or indirect role in terpenoid biosynthesis through multiple signaling pathways. AarbHLH47 interacted with TPS21 in the JA signaling network and might directly involve in the regulation of biosynthesis of β-caryophyllene, while AarbHLH48 was indirectly involved. AarbHLH8, 14, 21, 47 were indirectly involved in the regulation of key enzyme TPS-Cin of 1, 8-cineole biosynthesis through interacting with PHYB and PIF4 in the optical signal network, which had interaction with the upstream proteins HDS and DXR in the MEP pathway. However, two positive regulators, AarbHLH9 (homologous ID: AT1G22490) and AarbHLH34 (homologous ID: bHLH121), and a negative regulator, AarbHLH52 (homologous ID: UNE12) did not participate in the networks, and their functions need to be further explored.

## Discussion

Terpenoids are one of the most important pharmacologically active ingredients in *A. argyi*, and their content directly affects the medicinal quality and economic value of *A. argyi*. However, the biosynthesis and regulation mechanism of terpenoids in *A. argyi* and the regulatory role of bHLH family genes have not been studied. In this study, 53 AarbHLH proteins were identified from the transcriptome data in *A. argyi*, and the protein physicochemical properties were systematically analyzed, which is helpful for us to understand the bHLH family genes and their characteristics. It has been reported that there are 59 bHLH genes in highbush blueberry (Song et al., 2017) and 62 bHLH members in *Dracaena cambodiana* (Zhu et al., 2020) from the transcriptome level, which was equivalent to the number of bHLH genes identified in this study. Since the transcriptome data are not comprehensive in this study, and the genome of *A. argyi* has not been published, some bHLH family were still might not be identified yet. Based on genome data, 147 bHLH genes in *Arabidopsis thaliana* (Toledo-Ortiz et al., 2003), 121 bHLH genes in pineapple (Aslam M. et al., 2020) and 174 bHLH genes in sorghum (Fan et al., 2021) were identified. Therefore, the identification of bHLH family members needs to be improved by genomic data. According to the classification of bHLH family in *Arabidopsis thaliana*, the AarbHLH proteins were divided into 15 subfamilies, of which, XII subfamily had the largest numbers, which was consistent with that in *Arabidopsis thaliana*. However, eight subfamilies in *Arabidopsis thaliana* are not found in *A. argyi.* This may be due to the incompleteness of gene identification or difference in gene evolution. The analysis of the bHLH characteristic domains and conserved motifs indicated that all AarbHLH proteins contained the conserved bHLH domain, which was essential to bind target DNA and activate gene transcription. Even AarbHLH12, which lacked part of the amino acid sequence of the Helix2 region, still had conserved Leu-23 and Leu-56 amino acid positions for the function of the HLH dimer. In addition, proteins in the same subfamily usually had similar conserved motifs to perform specific functions. For example, MYC subfamily proteins contained a bHLH domain and a conserved bHLH-MYC_N domain. Studies have shown that the bHLH-MYC_N domain interacts with the JAZ protein in response to the JA signal (Chen S. K. et al., 2019).

bHLH TFs have been proven to be involved in terpenoid biosynthesis in several species, such as *Artemisia annua*, *Salvia miltiorrhiza*, and *Taxus wallichiana*. Thus, in this study, we further explored the functions of AarbHLH proteins in *A. argyi* and their regulation roles in terpenoid biosynthesis. The GO and KEGG analysis revealed that bHLHs participated in the biosynthesis of secondary metabolites and some biological processes, including signal transduction, plant development control, and metabolism in response to biotic and abiotic stresses. According to research reports, the volatile terpenoids in *A. argyi* were mainly accumulated in the leaves (Cao et al., 2017). Additionally, exogenous MeJA can also increase the biosynthesis and accumulation of terpenoids in most plants (Tugizimana et al., 2015; Ji et al., 2019). Therefore, GC-MS was used to determine the changes of 1, 8-cineole and β-caryophyllene, the main terpenoids in *A. argyi*, in the LSR group (in different tissues) and the MeJA group. The result showed the contents of 1,8-cineole and β-caryophyllene were the highest in the leaves and increased after MeJA treatment. On the other hand, the RNA-seq data of the LSR group and the MeJA group were used to analysis gene expression profiles of *AarbHLH* genes. The result of heat maps indicated that the repeatability of some *AarbHLH* genes in the MeJA group were not very well among three replicates (Figure 6B). This may be due to individual differences between sampling replicates although we have tried to choose the seedlings with uniform growth, or caused by technical errors in the process of RNA-seq library construction. Therefore, we used the average of FPKM value to analyze the gene expression differences. Through a comprehensive analysis of DEGs and homologous genes, and expression verification by qRT-PCR, 11 positive *TPS-AarbHLH*s and one negative *TPS-AarbHLH* that may be involved in terpenoid biosynthesis in *A. argyi* were screened. The correlation analysis also indicated that there 12 candidate *TPS-AarbHLHs* had significant correlations with the content of change of 1,8-cineole or β-caryophyllene in *A. argyi*.

The PPI networks were used to identify the interaction among TPS-AarbHLHs and terpenoid biosynthesis enzyme proteins (Braun et al., 2013). Our result revealed that nine TPS-AarbHLHs (AarbHLH8, 11, 14, 16, 21, 24, 28, 47, and 48), JA signal proteins, light signal proteins, and GA signal proteins formed a signal transmission network. These factors have also been proved to be essential signal factors involved in terpenoid biosynthesis (Zhang and Bjorn, 2009; Li et al., 2012). MYC2 and MYC4 were reported as important regulators in JA signaling pathway and terpenoid biosynthesis pathway. For instance, AaMYC2 was a JA-responsive factor and had positive regulation in artemisinin biosynthesis in Artemisia annua (Shen et al., 2016). In *Arabidopsis thaliana* and *Freesia hybrida*, the MYB-bHLH complex formed by MYC2 and MYB21 activated the sesquiterpene synthase TPS21 under the action of JA signal, and directly controlled the levels of β-caryophyllene or other sesquiterpenes (Picazo-Aragones et al., 2020; Yang et al., 2020). MYC4 acted additively with MYC2 in the activation of JA responses (Fernandez-Calvo et al., 2011). PIF proteins were positive regulators of the MEP pathway in *Arabidopsis thaliana*, and their overexpression synergistically up-regulated the synthetase genes of the MEP pathway, provided sufficient PPI precursors for the biosynthesis of monoterpenoids (Mannen et al., 2014; Chenge-Espinosa et al., 2018). In our network, AarbHLH47 and AarbHLH48 were identified as MYC-like proteins, which had 49.2 and 65.4% similarity with the MYC2 and MYC4 proteins in *Arabidopsis thaliana*, respectively (Supplementary Table S5), and may have interaction with β-caryophyllene synthase TPS21 (Figure 9). AarbHLH8 and AarbHLH14 were homologous PIF proteins. The correlation analysis result also indicated that these four genes had significant positive correlations with 1,8-cineole or β-caryophyllene, which supported the results of PPI networks. Therefore, AarbHLH8, 14, 47, 48 were considered as more likely positive regulators of terpenoid biosynthesis in *A. argyi* and their specific functions were worth to be further studied. In addition, AarbHLH11 and AarbHLH16 also participated in the regulation of the JA network. Our correlation analysis results also showed *AarbHLH11* and *AarbHLH16* had a significant positive correlation with 1,8-cineole and β-caryophyllene in the MeJA group (Table 3). Furthermore, although AarbHLH9, AarbHLH34, and AarbHLH52 were not predicted in the network, the results of differential expression and correlation analysis also predicted that they may be involved in the regulation of terpenoids biosynthesis. Of particular concern is AarbHLH52, which was the only possible negative regulator we screened. There were few reports on negative bHLH regulator of terpenoid biosynthesis were reported, therefore, its mechanism is also worthy of further study.

## Conclusion

In this study, 53 AarbHLHs were identified from the transcriptome of *A. argyi*. The systematic analysis of the physical and chemical features, proteins phylogeny, conserved motifs, and gene expression profiles of AarbHLHs has enriched the research of bHLH gene family in *A. argyi*. By comprehensive analysis of the content changes of targeted terpenoid active ingredients, the transcriptional expression profiles of *AarbHLHs* genes, the comparison of homologous functional proteins, and the prediction of PPI networks, 12 *AarbHLH* genes were screened for the first time that may be involved in terpenoids biosynthesis in *A. argyi*. This finding provides a basis for the functional identification of bHLH family genes and elucidation of the regulatory mechanism of bHLH TFs in the biosynthesis of volatile terpenoids in *A. argyi*, which will be of paramount significance for quality improvement in *A. argyi*.

## Data Availability Statement

The datasets presented in this study can be found in online repositories. The names of the repository/repositories and accession number(s) can be found below: NCBI SRA; PRJNA785536.

## Author Contributions

YS and SC contributed to conception of the study. XY drafted the manuscript, performed the qRT-PCR and metabolic experiment, edited and approved the final manuscript. XY, XW, and MW conducted the bioinformatics analysis. LY and LW contributed to validation and visualization. YS and XL revised the manuscript. All authors contributed to the article and approved the submitted version.

## Conflict of Interest

The authors declare that the research was conducted in the absence of any commercial or financial relationships that could be construed as a potential conflict of interest.

## Publisher’s Note

All claims expressed in this article are solely those of the authors and do not necessarily represent those of their affiliated organizations, or those of the publisher, the editors and the reviewers. Any product that may be evaluated in this article, or claim that may be made by its manufacturer, is not guaranteed or endorsed by the publisher.

## Footnotes

1
http://itak.feilab.net/

2
https://www.ncbi.nlm.nih.gov/orffinder/

3
http://pfam.xfam.org/

4
https://meme-suite.org/meme/tools/meme

5
http://cn.expasy.org/tools

6
http://www.genscript.com/psort/wolf_psort.html

7
http://kobas.cbi.pku.edu.cn/

8
https://www.genome.jp/tools/kaas/

9
https://www.ncbi.nlm.nih.gov/

10
https://string-db.org/

## Abbreviations

BP: biological processes
CC: cellular components
FAA: Folium *Artemisia argyi*
AAEO: *Artemisia argyi* essential oil GO Gene Ontology
HLH: helix loop helix
HMM: hidden Markov model
JA: jasmonic acid
KEGG: kyoto encyclopedia of genes and genomes
MF: molecular function
TF: transcription factor
TPS: terpenoid synthase
GC-MS: gas chromatography/mass spectrometry
DEGs: differently expressed genes
qRT-PCR: quantitative real-time PCR.
