# Supplementary material for: Integrated Analysis of Basic Helix Loop Helix Transcription Factor Family and Targeted Terpenoids Reveals Candidate AarbHLH Genes Involved in Terpenoid Biosynthesis in Artemisia argyi
Source: Front Plant Sci. 2022 Jan 17;12:811166. doi: 10.3389/fpls.2021.811166 (PMC8801783; doi:10.3389/fpls.2021.811166)
Supplement: Supplementary file 1 [file Data_Sheet_1.docx]

Supplementary Material

# Supplementary Figures and Tables

## Supplementary Figures


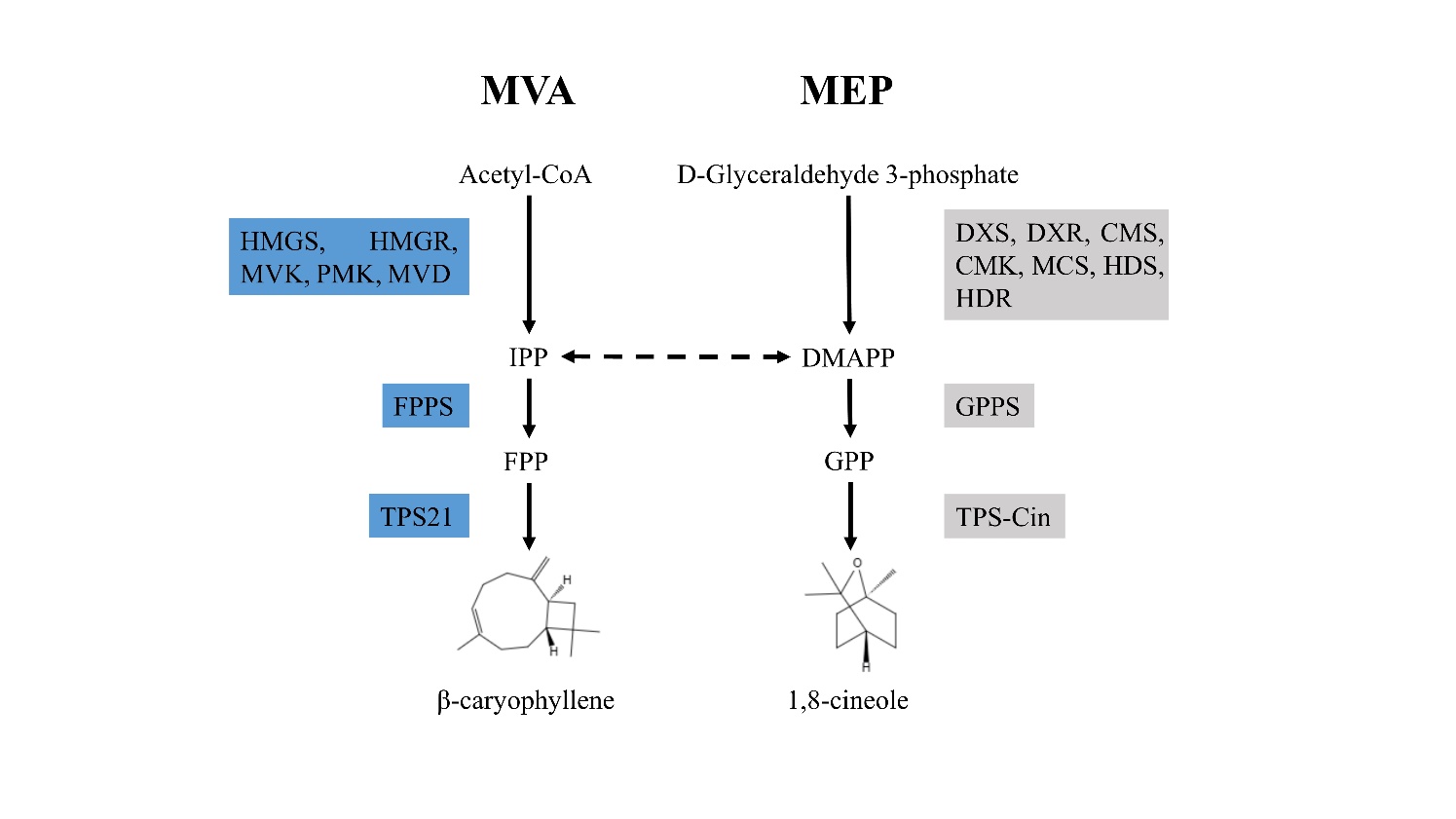


**Supplementary Figure S1.** Pathways leading to the production of 1, 8-cineole and *β*-caryophyllene.

Key enzyme genes in the MVA and MEP pathways are boxed in blue and gray. HMGS, hydroxymethylglutaryl-CoA synthase; HMGR, hydroxymethylglutaryl-CoA reductase; MVK, mevalonate kinase; PMK, phosphomevalonate kinase; MVD, diphosphomevalonate decarboxylase; FPPS, farnesyl diphosphate synthase; TPS21, beta-caryophyllene synthase; DXS, 1-deoxy-D-xylulose-5-phosphate synthase; DXR, 1-deoxy-D-xylulose-5-phosphate reductoisomerase; CMS, 2-C-methyl-D-erythritol 4-phosphate cytidylyltransferase; CMK, 4-diphosphocytidyl-2-C-methyl-D-erythritol kinase; HDS, (E)-4-hydroxy-3-methylbut-2-enyl-diphosphate synthase; HDR, 4-hydroxy-3-methylbut-2-en-1-yl diphosphate reductase; GPPS, geranylgeranyl diphosphate synthase; TPS-Cin, 1,8-cineole synthase.


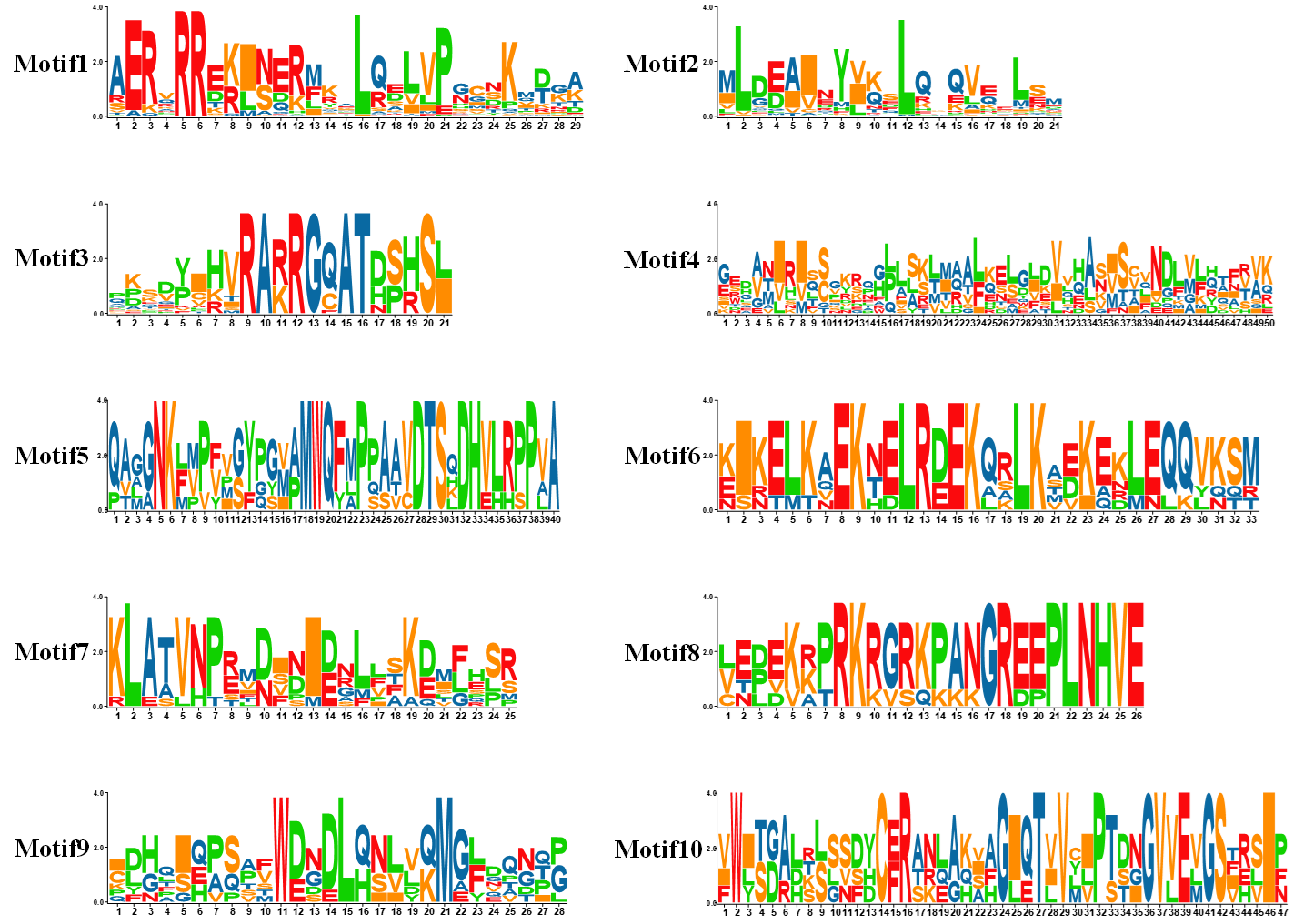


**Supplementary Figure S2.** The sequence logos of Motif 1~ Motif 10 in the MEME analysis.


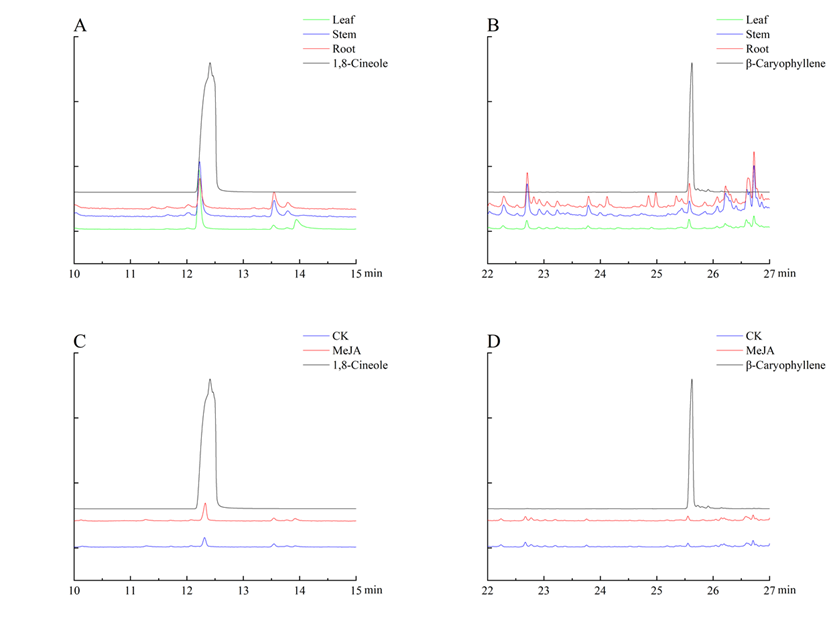


**Supplementary Figure S3.** Peak images of 1,8-cineole and *β*-caryophyllene samples by GC-MS

(A) Sample peaks of 1, 8-Cineole in the LSR group. (B) Sample peaks of *β*-Caryophyllene in the LSR group. (C) Sample peaks of 1, 8-Cineole in the MeJA group. (D) Sample peaks of *β*-Caryophyllene in the MeJA group.

**Supplementary Figure S4.** Comparative heat maps of *TPS-AarbHLHs* expression between RNA-Seq data and qRT-PCR in the LSR group and the MeJA group.

(A) The heat map of *TPS-AarbHLHs* expression of RNA-Seq data in the LSR group. (B) The heat map of *TPS-AarbHLHs* expression of qRT-PCR in the LSR group. (C) The heat map of *TPS-AarbHLHs* expression of RNA-Seq data in the MeJA group. (D) The heat map of *TPS-AarbHLHs* expression of qRT-PCR in the MeJA group.

## Supplementary Tables

**Supplementary Table S1.** List of qRT-PCR primers.

| **Gene** | **Specific primers (5’→3’)** | **Product length /bp** |
| --- | --- | --- |
| *AarbHLH7* | Forward: GCAGCCGCTCATCTCAGTCAAC | 107 |
|  | Reverse: GGAAGCGAGTTCATCATTGGGTCTT |  |
| *AarbHLH8* | Forward: TTTCGGGTCAAGCGGCTAATATGTC | 144 |
|  | Reverse: TCTGTGGAAGCGGTAATTGTGTCTG |  |
| *AarbHLH9* | Forward: GTGCTACACCTCAATGTCACCACA | 106 |
|  | Reverse: CACCTGCTATCTCGTCTGCTGAATT |  |
| *AarbHLH11* | Forward: CGGACGAGTGCTTGGTAAGAGATG | 103 |
|  | Reverse: TCCTCACGCCCATTAGCTGGTTTA |  |
| *AarbHLH14* | Forward: CGAGCCAGTTGTTGCTACTTCTTCT | 116 |
|  | Reverse: GGCACTCAAACTCCTCCGTATCAC |  |
| *AarbHLH16* | Forward: AATGGTGGTAATGCTGGTGGTAGTG | 110 |
|  | Reverse: TTCAACTTCTTCCTCCGTCGTCTCT |  |
| *AarbHLH21* | Forward: TCAAGAAGGGAAAGACAGGGAAGGA | 108 |
|  | Reverse: GCCGCTCCATAGATGTCATCCATG |  |
| *AarbHLH24* | Forward: TGAATTGGCGTCGATTCTTGAACCT | 110 |
|  | Reverse: GCTTCTGGGCTTCGTTTCTTAACTG |  |
| *AarbHLH28* | Forward: TCAGCCTAACGGTTATGGTGGAATG | 114 |
|  | Reverse: CCAGATGACGAACTCATTTGCCTCT |  |
| *AarbHLH34* | Forward: TGCCGATGCCACCTGGATCTATT | 103 |
|  | Reverse: AAAGCGGAAGCAGGATTGGAAACA |  |
| *AarbHLH47* | Forward: CCTCTACAGTTTCACCACCCGAAG | 101 |
|  | Reverse: GGCGTCCCACCCAATGATCTTG |  |
| *AarbHLH48* | Forward: GCAGCTAGGCTAATGGCAGTGTT | 101 |
|  | Reverse: TTCACAGTCGCTTGTTGGATCATCA |  |
| *AarbHLH52* | Forward: CCAGCCGATACCTAACACAGTGC | 120 |
|  | Reverse: CGACGAAGCCTCTCAGCAATACTAT |  |
| *Actin* | Forward: GTCACACTGGTGTCATGGTTGGAAT | 105 |
|  | Reverse: CGTGCTCAATCGGGTACTTCAAAGT |  |

**Supplementary Table S2.** Raw data of peak area of 1,8-cineole and *β*-caryophyllene in GC-MS.

|  | **Raw data of peak area** | |
| --- | --- | --- |
|  | **1,8-cineole** | ***β*-caryophyllene** |
| **Tissues** | **The LSR group** | |
| Leaf-1 | 19787810.10 | 1656267.73 |
| Leaf-2 | 20433317.22 | 1129987.39 |
| Leaf-3 | 20030352.69 | 1305965.42 |
| Stem-1 | 3068915.38 | 359420.79 |
| Stem-2 | 1682393.18 | 380383.29 |
| Stem-3 | 2507595.98 | 397154.43 |
| Root-1 | 1743422.92 | 662638.48 |
| Root-2 | 1236275.60 | 519003.97 |
| Root-3 | 1462547.62 | 629427.66 |
| **Treatments** | **The MeJA group** | |
| CK-1 | 519402.93 | 136089.14 |
| CK-2 | 682541.52 | 148809.45 |
| CK-3 | 600972.22 | 142449.30 |
| MeJA-1 | 828488.08 | 168151.68 |
| MeJA-2 | 1128655.49 | 177557.21 |
| MeJA-3 | 978571.79 | 172854.45 |

**Supplementary Table S3.** The FPKM raw data of *AarbHLH* genes in the transcriptome.

| **FPKM raw data** | | | | | | | | | |
| --- | --- | --- | --- | --- | --- | --- | --- | --- | --- |
| Gene name | Leaf | Stem | Root | CK1 | CK2 | CK3 | MeJA1 | MeJA2 | MeJA3 |
| *AarbHLH1* | 0 | 2.24 | 8.49 | 0 | 14.62 | 11.45 | 8.27 | 12.14 | 0 |
| *AarbHLH2* | 1.57 | 1.97 | 0.25 | 0 | 28.08 | 33.88 | 12.21 | 15.18 | 0 |
| *AarbHLH3* | 9.34 | 38.78 | 25.18 | 14.76 | 38.98 | 17.63 | 22.52 | 20.35 | 12.94 |
| *AarbHLH4* | 0.46 | 1.61 | 0.09 | 7.93 | 2.21 | 12.51 | 14.19 | 14.43 | 5.11 |
| *AarbHLH5* | 0.29 | 0.57 | 0 | 48.01 | 35.8 | 11.91 | 44.18 | 50.12 | 22.06 |
| *AarbHLH6* | 14.88 | 3.91 | 0 | 19.62 | 11.16 | 5.77 | 20.41 | 13.92 | 14.63 |
| *AarbHLH7* | 17.56 | 4.14 | 0.33 | 24.37 | 21.91 | 13.61 | 11.14 | 12.12 | 18.72 |
| *AarbHLH8* | 7.97 | 5.77 | 0.36 | 21.39 | 4.85 | 11.16 | 13.13 | 14.65 | 10.91 |
| *AarbHLH9* | 4.04 | 0.86 | 1.01 | 30.54 | 43.29 | 44.99 | 30.03 | 40.54 | 68.24 |
| *AarbHLH10* | 1.08 | 2.97 | 0.55 | 26.09 | 25.57 | 25.76 | 12.32 | 16.66 | 9.04 |
| *AarbHLH11* | 50.69 | 245.22 | 81.88 | 14.8 | 25.87 | 25.61 | 46.58 | 40.65 | 36.65 |
| *AarbHLH12* | 9.34 | 38.78 | 25.18 | 27.6 | 0 | 0 | 4.72 | 7.4 | 0 |
| *AarbHLH13* | - | - | - | 24.68 | 19.9 | 20.94 | 11.53 | 16.23 | 11.55 |
| *AarbHLH14* | 17.19 | 20.7 | 4.54 | 14.47 | 12.71 | 8.39 | 10.58 | 8.77 | 24.77 |
| *AarbHLH15* | 59.31 | 47.8 | 0.19 | 77.4 | 64.38 | 60.04 | 70 | 51.93 | 70.39 |
| *AarbHLH16* | 2.99 | 7.31 | 1.77 | 2.96 | 5.03 | 9.33 | 10.08 | 0 | 0 |
| *AarbHLH17* | 2.96 | 6.15 | 16.01 | 10.78 | 8.21 | 11.61 | 19 | 13.06 | 5.85 |
| *AarbHLH18* | 137.04 | 257.31 | 119.14 | 170.04 | 224.56 | 137.35 | 164.67 | 179.59 | 235.57 |
| *AarbHLH19* | 13.6 | 1.56 | 0.18 | 105.96 | 123.51 | 99.19 | 88.81 | 112.22 | 91.43 |
| *AarbHLH20* | 5.16 | 7.29 | 3.71 | 10.56 | 10.31 | 5.32 | 17.69 | 12.95 | 3.09 |
| *AarbHLH21* | 4.19 | 2.77 | 0.77 | 3.99 | 9.04 | 2.88 | 7.04 | 14.62 | 3.17 |
| *AarbHLH22* | 8.15 | 313.2 | 25.75 | 2.69 | 8.12 | 3.43 | 3.72 | 11.46 | 15.36 |
| *AarbHLH23* | 11.95 | 6.65 | 1.66 | 7.86 | 21.9 | 26.8 | 24.26 | 37.15 | 16.08 |
| *AarbHLH24* | 68.3 | 68.58 | 35.07 | 74.78 | 48.64 | 65.7 | 101.33 | 112.99 | 85.51 |
| *AarbHLH25* | 0.74 | 2.72 | 0.17 | 17.08 | 10.75 | 10.93 | 22.57 | 17.59 | 17.27 |
| *AarbHLH26* | 6.07 | 19.12 | 0.09 | 34.44 | 43.42 | 29.34 | 20.49 | 16.01 | 36.64 |
| *AarbHLH27* | 25.77 | 44.3 | 85.06 | 61.22 | 36.58 | 45.28 | 55.13 | 67.12 | 54.21 |
| *AarbHLH28* | 3.83 | 1.93 | 0.64 | 6.12 | 6.67 | 7.76 | 5.72 | 19.84 | 5.79 |
| *AarbHLH29* | 9.47 | 15.39 | 3.03 | 18.63 | 9.23 | 10.15 | 11.92 | 13.02 | 2.44 |
| *AarbHLH30* | 19.55 | 16.74 | 7.9 | 92.63 | 69.92 | 57.82 | 27.48 | 41.68 | 83.26 |
| *AarbHLH31* | 4.01 | 15.72 | 6.58 | 43.45 | 26.91 | 21.35 | 36.04 | 38.74 | 17.16 |
| *AarbHLH32* | 11.99 | 17.24 | 3.58 | 4.94 | 1.26 | 7.31 | 2.43 | 3.95 | 0 |
| *AarbHLH33* | 0.77 | 2.27 | 0.67 | 7.16 | 10.57 | 13.09 | 8.05 | 0 | 6.99 |
| *AarbHLH34* | 11.55 | 8.84 | 4.29 | 5.77 | 10.77 | 13.01 | 10.82 | 9.14 | 11.03 |
| *AarbHLH35* | 0.16 | 2.92 | 0 | 19.84 | 8.16 | 6.05 | 8.22 | 2.61 | 11.31 |
| *AarbHLH36* | 37.57 | 30.41 | 25.06 | 26.64 | 17.52 | 51.81 | 58.51 | 55.34 | 25.59 |
| *AarbHLH37* | 12.67 | 8.87 | 3.61 | 38.19 | 36.82 | 58.27 | 64.39 | 40.99 | 28.71 |
| *AarbHLH38* | 16.42 | 16.7 | 10.2 | 8.36 | 12.91 | 39.09 | 42.86 | 16.61 | 24.11 |
| *AarbHLH39* | 12.39 | 4.66 | 1.2 | 90.46 | 87.25 | 78.02 | 87.26 | 66.68 | 103.59 |
| *AarbHLH40* | 103.15 | 89.19 | 20.07 | 44.08 | 61.33 | 77.71 | 53.52 | 71.68 | 55.71 |
| *AarbHLH41* | 3.88 | 6.35 | 0.53 | 55.05 | 50.62 | 44.6 | 26.92 | 43.77 | 30.82 |
| *AarbHLH42* | 1.8 | 5.64 | 0.33 | 8.27 | 14.79 | 10.23 | 18.55 | 14.61 | 15.74 |
| *AarbHLH43* | 47.7 | 78.36 | 21.8 | 39.79 | 74.97 | 72.27 | 69.21 | 54.06 | 47.86 |
| *AarbHLH44* | 1.12 | 2.98 | 1.25 | 18.51 | 10.46 | 7.41 | 23.2 | 36.92 | 10.78 |
| *AarbHLH45* | 64.44 | 145.9 | 59.4 | 17.05 | 23.15 | 9.48 | 21.79 | 20.04 | 22.15 |
| *AarbHLH46* | 17.24 | 11.97 | 5.63 | 62.98 | 59.35 | 54.57 | 34.67 | 48.01 | 48.59 |
| *AarbHLH47* | 20.95 | 10.83 | 9.24 | 31.34 | 20.07 | 17.19 | 26.29 | 29.73 | 14.56 |
| *AarbHLH48* | 565.21 | 443.06 | 406.92 | 54.71 | 40.53 | 47.38 | 78.11 | 68.86 | 85.26 |
| *AarbHLH49* | 0 | 0.23 | 0.45 | 29.75 | 15.55 | 13.15 | 10.79 | 17.74 | 0 |
| *AarbHLH50* | 1.3 | 10.24 | 0 | 62.44 | 23.5 | 68.86 | 25.91 | 16.83 | 104.3 |
| *AarbHLH51* | 11.59 | 8.42 | 1.43 | 30.89 | 26.03 | 18.77 | 25.82 | 31.58 | 45.19 |
| *AarbHLH52* | 4.6 | 17.91 | 11.55 | 6.99 | 0 | 8.92 | 4.66 | 2.92 | 6.39 |
| *AarbHLH53* | 0 | 2.23 | 0.75 | - | - | - | - | - | - |

**Supplementary Table S4.** BLAST alignment information for TPS-bHLH proteins and TPS-AarbHLH proteins.

| **TPS-bHLHs** | **TPS-AarbHLHs** | **Total Score** | **E value** | **Percent identify (%)** |
| --- | --- | --- | --- | --- |
| AtMYC2 | AarbHLH47 | 547 | 0.0 | 49.24 |
|  | AarbHLH48 | 298 | 8e-98 | 48.82 |
| AtPIF5 | AarbHLH7 | 165 | 2e-48 | 35.41 |
|  | AarbHLH8 | 136 | 7e-39 | 59.69 |
|  | AarbHLH14 | 177 | 1e-36 | 63.37 |
| AabHLH1 | AarbHLH24 | 389 | 1e-140 | 95.69 |
| AabHLH112 | AarbHLH16 | 492 | 2e-178 | 92.18 |
| AaPIF3 | AarbHLH14 | 1124 | 0.0 | 96.62 |
| AaMYC2 | AarbHLH47 | 1244 | 0.0 | 97.13 |
| SmMYC2 | AarbHLH47 | 613 | 0.0 | 52.21 |
|  | AarbHLH48 | 332 | 2e-111 | 49.64 |
| SmbHLH10 | AarbHLH34 | 335 | 6e-118 | 62.50 |
| SlMYC1 | AarbHLH47 | 643 | 0.0 | 53.58 |
|  | AarbHLH48 | 367 | 1e-124 | 55.75 |
| CrBIS1 | AarbHLH1 | 89.4 | 4e-24 | 26.92 |
| CrBIS2 | AarbHLH1 | 99.0 | 3e-27 | 32.98 |
| CrBIS3 | AarbHLH1 | 95.5 | 3e-26 | 31.79 |
| PbbHLH4 | AarbHLH16 | 318 | 6e-109 | 55.13 |
|  | AarbHLH2 | 272 | 2e-92 | 76.76 |
| BpbHLH9 | AarbHLH9 | 214 | 3e-69 | 54.76 |
|  | AarbHLH53 | 198 | 4e-64 | 42.39 |
| CubHLH3 | AarbHLH1 | 110 | 1e-31 | 35.16 |
| CpBHLH13 | AarbHLH11 | 477 | 4e-167 | 48.79 |
|  | AarbHLH45 | 408 | 9e-141 | 50.76 |
| DobHLH4 | AarbHLH16 | 322 | 2e-110 | 55.79 |
|  | AarbHLH2 | 270 | 1e-91 | 76.22 |
| MtTSAR1 | AarbHLH1 | 84.3 | 2e-22 | 30.91 |
| MtTSAR2 | AarbHLH1 | 95.1 | 2e-26 | 31.84 |

**Supplementary Table S5.** Candidate TPS-AarbHLHs and their homolog proteins in *Arabidopsis thaliana*.

| **AarbHLH protein** | **Homolog AtbHLH protein** | **Identity （%）** | **Function** |
| --- | --- | --- | --- |
| AarbHLH8 | PIL5 | 41.6 | Phytochrome interaction factor |
| AarbHLH9 | AT1G22490 | 56.7 | Transcriptional regulation factor |
| AarbHLH11 | AIB | 49.1 | ABA-inducible BHLH-type transcription factor |
| AarbHLH14 | PIF3 | 38.7 | Phytochrome interaction factor |
| AarbHLH16 | ICE1 | 62.3 | Encodes a MYC-like bHLH transcriptional activator |
| AarbHLH21 | BIM1 | 38.9 | Brassinoid steroid signal regulation |
| AarbHLH24 | ILR3 | 58.8 | Responses to metal steady-state changes |
| AarbHLH28 | FBH4 | 47.5 | Transcriptional regulation of flowering |
| AarbHLH34 | bHLH121 | 39.3 | Responses to metal ions such as Ca+ and K+ |
| AarbHLH47 | MYC2 | 49.2 | Light, ABA, and JA signals regulate transcription factors |
| AarbHLH48 | MYC4 | 65.4 | JA signals regulate transcription factors |
| AarbHLH52 | UNE12 | 60.2 | Required for ovule fertilization |

The identity values and protein functions were derived from STRING.
